# A qualitative study of professional and client perspectives on information flows and decision aid use

**DOI:** 10.1186/1472-6947-12-26

**Published:** 2012-03-29

**Authors:** Christine Stirling, Barbara Lloyd, Jenn Scott, Jenny Abbey, Toby Croft, Andrew Robinson

**Affiliations:** 1 Private Bag 135 School of Nursing and Midwifery, Hobart University of Tasmania, Hobart, Australia; 2 Wicking Dementia Research and Education Centre, Private Bag 23 17 Liverpool Hobart, St, University of Tasmania, Hobart, Australia; 3 Private Bag 30, School of Psychology, University of Tasmania, Hobart, Australia; 4 Wicking Dementia Research and Education Centre, University of Tasmania, Hobart, Australia; 5 Department of Psychology, Liverpool St, Hobart, Royal Hobart Hospital, Hobart, Australia; 6 School of Nursing and Midwifery, Private Bag 121 Hobart, University of Tasmania, Hobart, Australia; 7 Private Bag 121, Wicking Dementia Research and Education Centre, University of Tasmania, Hobart, TAS, Australia 7000

**Keywords:** Aged care, Carers, Decision making, Dementia, Qualitative

## Abstract

**Background:**

This paper explores the meanings given by a diverse range of stakeholders to a decision aid aimed at helping carers of people in early to moderate stages of dementia (PWD) to select community based respite services. Decision aids aim to empower clients to share decision making with health professionals. However, the match between health professionals' perspectives on decision support needs and their clients' perspective is an important and often unstudied aspect of decision aid use.

**Methods:**

A secondary analysis was undertaken of qualitative data collected as part of a larger study. The data included twelve interviews with carers of people with dementia, three interviews with expert advisors, and three focus groups with health professionals. A theoretical analysis was conducted, drawing on theories of 'positioning' and professional identity.

**Results:**

Health professionals are seen to hold varying attitudes and beliefs about carers' decision support needs, and these appeared to be grounded in the professional identity of each group. These attitudes and beliefs shaped their attitudes towards decision aids, the information they believed should be offered to dementia carers, and the timing of its offering. Some groups understood carers as needing to be protected from realistic information and consequently saw a need to filter information to carer clients.

**Conclusion:**

Health professionals' beliefs may cause them to restrict information flows, which can limit carers' ability to make decisions, and limit health services' ability to improve partnering and shared decision making. In an era where information is freely available to those with the resources to access it, we question whether health professionals should filter information.

## Background

Shared decision making between health professionals and clients is now recognized as an imperative for improving primary health care outcomes [1]. Decision support needs and factors facilitating decision partnerships are, however, complex and contextual and it is increasingly clear we need to better understand this complexity [2-6]. Decision aids (DAs) are known to help individuals to make health care choices in complex situations, and when outcomes may be indeterminate or dependent on values and beliefs [7]. However, many research questions remain to be answered as to how DAs work in different settings, and in particular what influence diverse professional cultures might exert on the success or otherwise of decision aids targeting health service consumers [6,8]. This paper makes a contribution to addressing this research deficit, by reporting on a project which uncovered connections between the way three groups of health service providers 'positioned' carers and their perceptions of carers' decision support needs.

### Care choices for carers of people with dementia

This paper emerges from a larger DA development and piloting study (reported elsewhere [9]) aimed at helping carers of people in early to moderate stages of dementia (PWD) to select community based respite services. There are three main forms of respite - in the home respite, adult day care respite and overnight institutional based care. All are aimed at decreasing carer burden and have the potential to delay institutionalisation for the person with dementia [10,11]. In spite of potential benefits, however, respite is underutilized by carers. A complex range of socio-cultural factors are implicated in carer 'reluctance to use services' in general [12] and carers may need support to weigh up available options. Despite their vulnerability to stress-related conditions such as depression [13], we argue that access to realistic, contextually relevant information is an essential component of informed service-related decision making for dementia carers.

The findings presented here are particularly interesting in the light of recent attention to the wider contexts that influence the implementation of DAs [5,6]. As the care-recipient's disease progresses, carers of PWD are required to take increased responsibility for service decisions. Dementia is an incurable ageing-related condition which generates high levels of disability [14]. As such, it imposes a great burden of care on the health system of ageing societies. The effect on family carers can also be devastating, with unrelieved care giving, often resulting in psychological, physical, financial, and social stress [13,15,16]. The problem is growing, with estimates of a doubling of the numbers of PWD in Australia by 2030 [14]. Decision support to help carers select services that will improve their capacity to provide care in the home is an important strategy for limiting the impacts of the illness on PWD, their carers and the health system.

DAs help synthesize information and preferences in order to facilitate informed and person-centred health care decisions. They can improve knowledge, lower decisional conflict, reduce indecision, increase patient decision making involvement, and promote realistic expectations regarding outcomes [7]. DAs therefore have the potential to reduce the confusion and knowledge deficits that carers experience as they attempt to participate in complex health care decisions. While research has begun to address dementia carers' information needs during late stages of dementia see [17], for example, this is the first DA we are aware of that targets services that carers' can use during the early to moderate phases of dementia. The DA [18] has a typical structure containing brief information about the types of common community services available. This is conceptualized as: learning about your situation, getting help with particular problems, getting help if you have too much to do, and getting time to recharge your batteries. We then focus on describing respite care and provide decision tools based on selecting a respite care option, including step by step 'weigh scales' (adapted from [19] with permission), and telephone numbers and links to facilitate gaining further information. The DA also contains vignettes describing carers' experiences of increasing burden as their relative deteriorates, and brief targeted information about the trajectory of decline in dementia, which was considered information that was potentially stressful for carers.

The service choices of individual carers can be considered 'evaluative', in that the decisions of each individual will be a result of factors that stem from their social, economic, cultural and personal situations. Factors such as caregiver resources, the tasks of care (such as managing the behavioural and psychosocial symptoms of the care-recipient), and carers' attributes, will influence carers' evaluations of their situation [20-22]. Carers' interactions with health professionals will also influence how they perceive their own situations, the resources they are given, and the choices open to them [23]. Every care decision is therefore heavily context dependent.

Wackerbarth [24] found that many carers of PWD had difficulty making care decisions. In general, they preferred to make small care-related changes when they judged a situation to be deteriorating. Judgements were very individual, as all carers demonstrated different levels of tolerance for their situations. Nevertheless, many carers engage with services in response to crises, at which point they are less able to think through decisions. Carers are known to vary in their desire for and response to information, but carers of PWD often want more information, and closer to diagnosis [17]. Knowledge of the facts of dementia is associated with decreased rates of depression, more realistic expectations, increased feelings of competence, and the increased use of positive comparisons in carers [17,25,26]. However it is also associated with some anxiety [17,26], with Chang et al. [17] finding that 51% of carer respondents felt some anxiety when reading sections of their dementia information booklet, but only 11% felt it was too confronting.

### Professional cultures, positioning and shared decision-making

While long advocated for health professionals, shared decision-making, in which clinicians and clients participate together at all stages of the decision-making process, has not been widely adopted in practice [6]. Although the beliefs and actions of health professionals can impact significantly on interactions aimed at joint decision-making with carers, few studies have acknowledged the inherent power imbalances within the health system through which this occurs [4,23]. The positioning of various stakeholders is also an unexplored element in decision support. Attention to the ways in which health service clients are positioned by service providers, and the ways in which clients position themselves, can help to shed light on the dynamics of power relations in the context of health service provision.

The term 'position' refers to "a metaphorical location taken in the psychological space afforded a person in a particular social episode or clinical conversation, whereby any participant may publicly claim more or less right, responsibility or duty to act" [27], page 62. It is the viewpoint from which people identify themselves or others with certain groups or politics, or to the meanings they apply to technologies and interventions [27]. Within particular contexts, health professionals and clients will 'position' themselves, and be positioned by others. Life circumstances influence the positions available to individuals and groups, and positioning is one way that more powerful groups can control what it is 'possible' to know. Attention to positioning is important, because it will influence how carers and health professionals interact with each other and with any new technologies. One study found that carers are typically positioned in one of four ways: as free resources to support health workers, as co-workers working with professionals, as co-clients who need support themselves, or as caregivers superseded by professional interventions [28]. The way in which health professionals position carers will accordingly influence their decision making interactions with carers and the degree of agency they ascribe to carers.

The act of positioning may be deliberate or unconscious, and is shaped by the expectations of self, others, or role identities [27], page62. Members of particular professional groups are socialized as to the appropriateness of certain orientations towards clients [29]. The more they enact those orientations, the more invested they become in maintaining them, but clients may not feel empowered to request alternative approaches that resonate more with their needs [30]. Health professionals' well-intentioned positioning of clients as vulnerable and in need of protection may thus have unintended negative consequences for service recipients.

Health professionals are favorably positioned as specialized information givers in care settings [23]. They are powerful in this situation and can influence information flows in a manner which may or may not benefit health consumers [2,23]. Nurses, for example, can be reluctant to share knowledge and decision making control [4], and it has been suggested that health professionals may withhold information [17]. One study found a considerable gap between physicians intention to use a DA and actual use [8]. A review of the literature on health professionals' perceptions found that lack of time and lack of relevance in the clinical setting were the most frequently mentioned obstacles to shared decision making [6]. However a range of other barriers suggested to the review authors that 'health professionals might be screening *a priori' *patients they consider eligible [6], page 5. This is concerning because not all clients have equal access to resources and health professionals may misjudge clients' information needs or preferences [6].

Carers' own experiences of empowerment in decision making within the community setting is not yet fully understood. While carers feel they control the entry of some professional services into their homes [31], the presence of other services can generate feelings of loss of control [32]. In the acute setting, carers have complained of feeling excluded from decision making [33]. A need for further understanding carers' perceptions of their agency in decision making is therefore indicated.

The beliefs of particular groups about carers are structured by assumptions that shape consequent action [34]. We accordingly consider issues of interaction between service providers and recipients by exploring the meanings given to decision support for dementia carers by a diverse range of stakeholders. Particular attention is paid in the analysis to the 'positioning' of carers by representatives of key stakeholder groups, namely dementia carers, expert advisors and health professionals. The paper highlights connections between beliefs about carers and beliefs about information control in decision support for health care consumers.

## Methods

A supplementary secondary analysis was conducted of qualitative interview and focus group data collected during the development of the DA [9]. Secondary analysis of qualitative data allows material collected for another purpose to be 'mined' for alternative meanings, in ways that complement or transcend the original study see [35]. Supplementary secondary analysis extends the scope of the original project, with "a more in-depth focus on an emergent issue or aspect of the data which was not addressed, or was only partially addressed, by the primary research" [35], pages 41-42.

Ethics approval was granted for the original development study by the Tasmanian Social Science Human Research Ethics Committee. For that study, we sourced a convenience sample of 13 experienced carers (caring for more than 3 years) from previous unrelated studies and key support organizations. Our carer participants had characteristics consistent with the demographic profile of carers of PWD in Australia [14] though females and spouses were slightly over-represented. Most participants were female (85%), the spouse of the care recipient (85%) and aged over 66 (77%). Face-to-face interviews took between one and two hours. Questions focused on eliciting participants' perceptions of the usefulness, content and style of the DA, and of their own information and decision making needs. An advisory panel of community service providers included representatives from three key umbrella groups in the field of dementia, one of which focused on the disease itself, one on carers and one on respite services. These advisors provided access to 'expert' voices on dementia care. Expert panel participants were asked to review the DA and to contribute their perspectives during qualitative interviews.

In addition, three audiotaped focus groups were conducted with health care professionals from three community health service providers. The aim was to understand if and how different providers might use the DA and to evaluate which components might improve uptake of the intervention. The resulting 12 voluntary participants were mostly female (11 females: 1 male) and had an average age of 50. They included four community nurses, four counselors, and four community support workers. Due to the large amount of irrelevant data in the transcripts notes were extracted from these tapes by the researcher, after consultation and consensus with the chief investigator. It was during this process that the research team first identified issues around the positioning of carers.

**Figure 1:**
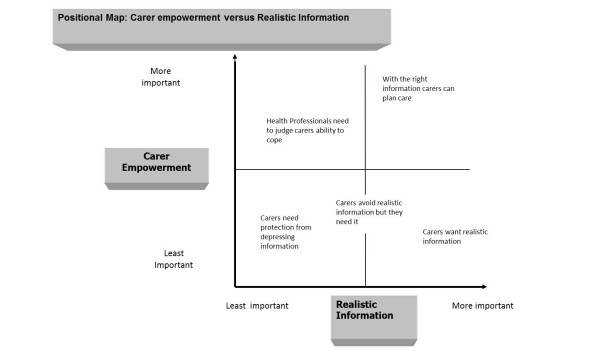
**Positional map of carer empowerment versus realistic information**.

Data was analysed, applying Clark's [36] method of positional mapping, by two members of the research team. Positional maps 'lay out the major positions taken and *not *taken, in the data vis-à-vis particular discursive axes of variation and difference... surrounding complicated relational issues in the situation.' [36] page 554. The researchers explored rival explanations, probed biases, and clarified the basis of interpretation, in order to enhance the credibility of the analysis. Common themes that emerged in reaction to the decision aid were: situating carers as empowered or passive, disagreements over carers' need for realistic information, and disparate perspectives as to whether the DA tool was useful for carers and/or health professionals. Positional maps were developed to highlight the major positions taken by research participants in relation to carers and the DA. These analytic maps represent the range of positions taken on particular issues in the data, using two axes of positions on a continuum. We found, for example, that carers were positioned as empowered (on a continuum of more or less), and that this related to the a second axis of carers' need for realistic information (on a continuum of more or less) (See Figure 1). In the following section we describe the key positions and relationships that emerged from the qualitative data.

## Results

### Positioning carers

There were key differences amongst the informants regarding the type of information they believed was appropriate for carers. In Figure 1 we map these varied beliefs about carers against key differences in views on carers' empowerment. One axis covers the positioning of carers as empowered on a continuum of more or less, and the second axis covers the positioning of carers' need for realistic information on a continuum of more or less.

Four positions were uncovered in the qualitative data. A key position located the carer as an empowered subject needing realistic information as a crucial resource for managing his or her situation and planning for the future. This position was adopted by carers (with one exception) and community nurses. One carer stated that exposure to confronting stories was a necessary aspect of decision support: *'You need to put confronting stories in. You realize as you go along that it's not going to get any better. You need to face reality. I had to wash my toilet six times a day. It's hard!' (Carer 3)*. An important element of realistic information for carers was that the DA reinforced the fact that dementia had a trajectory of decline and included stories about the increasing care demands this deterioration brings: *'The [DA] information is good because they point out that it is never going to get better. That's an important message.' (Carer 5)*.

The second position took a more paternalistic view of carers, situating them as needing to be protected from realistic information. Carers were positioned as unable to cope with upsetting realities, and the assumption was that realistic information, such as the stories of carers being stressed by behaviors of PWD such as repetitive questioning and wandering, was likely to cause them to feel depressed. This perspective was clearly expressed by an expert advisor: '*The Gold Book decision aid is too confronting for carers' (Expert Advisor 1)*. In this positioning, participants felt that the timing of information giving was important and that they themselves were able to judge the 'best time' for information. One support worker expressed this succinctly: '*Not everyone is ready for this sort of thing. It needs to be given at the right time' (Support Worker 1)*. Expert advisors, counsellors and support workers were most likely to use this positioning, but only one carer expressed this view.

The third position situated carers as passive, but needing realistic information, which would compel them to see and plan for the future. '*Carers don't want to plan for the future, they sometimes cover their ears. Then service use typically starts from a crisis' (Expert Advisor 2)*. The final position evident in the data was a more nuanced one. This situated carers as partially empowered, with the level of information needed by carers dependent on the individual situation. Again however, the health professional was positioned as needing to judge how or when carers might want to access the information. The following quote illustrates the perspective of one counselor: '*Once the mind is free of all that then they can begin problem solving. If you bring in a service too early when they haven't sorted all those psychological factors then there will be problems (Counselor 2).' *While this belief exhibits elements of paternalism, carers are acknowledged as capable of decision making under the right circumstances, as judged and facilitated by the counselor.

The positioning of carers on a continuum of more or less empowered was related to views on how much realistic information carers should be given. While positions on a map should not be ascribed to any one group or individual [36], there were some clear differences between health professionals/workers and carers. Counselors, support workers and expert advisors were likely to position carers more paternalistically, suggesting that carers needed information to be provided 'when ready', and in a 'softened' or 'protective' format. Too much realistic information was represented as likely to generate despair, depression, or an inability to cope. Instead carers were positioned as needing to be gently assisted in a staged manner to come to terms with the future deterioration and death of the care-recipient, and services were viewed as a means to support them through this process. This position implied that the experts would be able to judge 'when' carers needed information, and the 'type' of information they would need. Carers and community nurses, on the other hand, were likely to represent carers as empowered individuals who require realistic information in order to plan and decide about services. These differences highlight the ability of professionals' beliefs to affect carers' access to resources.

### Positioning the DA

Data supported a second positional map which mapped participants' views on whether the DA was useful for carers, and secondly whether the DA was useful as a tool for health professionals.

Many participants felt that carers would benefit from the DA. For some - mostly carers and community nurses - the DA was useful to carers as a stand-alone item. The following quote details one community nurse's perception that the DA provided information that helped carers negotiate the complex community service sector: '*There are so many services out there, so many way of receiving services, and this really captures that, the advantages and disadvantages. It's really great; it just puts it in a nutshell.' (Community Nurse 2)*. For carers, the way the decision aid highlighted the trajectory of dementia was also seen as very valuable, as seen in this career's quote: '*I really feel every carer needs one, it gives you the things you need to know and it's an ongoing thing, dementia, and if you have a booklet like this it gives you some idea of what is available' (Carer 3*). Carers related strongly to the vignettes, which in some cases, normalized their experiences to the extent that relieved them from guilt over respite decisions: '*If I had had this DA when I first made the decision to put my husband in respite care, it would have made me feel better about the decision' (Carer 8)*. Several carers expressed regret that they had not been able to access the DA earlier in their own caring experiences, as reflected in the following quote: '*If I had done the carer stress test earlier I would have sought help earlier' (Carer 6)*. Overall, the majority of carers and community nurses expressed a strong belief that the DA was useful to carers.

A second key position was that the DA would be useful for health professionals or others to work through with carers. This positioned the DA as useful to carers, both directly for personal use and indirectly as a tool for carer education and counseling. The DA was perceived as potentially useful as a discussion point with carers, as a take-away prompt for carers, and as a counseling device for the health worker. One role proposed for the DA was as a form of 'back-up' information to leave behind after discussion with carers. As this community nurse pointed out, carers cannot always retain large amounts of new information: '*You can talk to someone, and they can be really engaged about their decisions, but when you walk out the door they can go blank. But if we leave something like this behind then they can relook at it and we can talk about it next time when we come' (Community Nurse 2)*. Some carers saw the value of the DA to be used in a peer support capacity. One carer suggested that it could serve as a valuable referent at peer support meetings: '*The Gold Book decision aid could be used in a group situation so more experienced carers could give new carers support as they work through the book. At that time you only have your own thoughts and you're isolated so much. This would be a very good idea to bring to the carers meeting' (Carer 6)*.

A less commonly asserted perspective was that the DA was not useful. This was linked to the passive positioning of carers as co-clients needing to be protected from information. The following quote shows how an expert advisor was certain that carers did not need more information: '*Carers don't want booklets, don't want information, they are already stressed by too much written information given to them by X organization.' (Expert Advisor 3)*. Some participants considered the book 'not useful' for carers, but also 'not useful' as a tool for particular health professionals. As this counselor stated: '*The DA is not suited for how we work because we operate on forming a connection with people [carers] which they seem to like and need' (Counselor 1)*. These beliefs are in direct opposition to the perspectives expressed by all but one carer, but they were associated with particular roles. This highlights the importance of understanding health worker norms in DA interventions.

## Discussion

Disparate positioning of carers is evident in the expressed perspectives of carers, health professionals and expert advisers. The positioning of carers as either empowered or passive was clearly linked to beliefs about the appropriateness of providing realistic information and the type of support that carers need when making decisions. In turn, these beliefs appeared to be located within professional roles and ideologies of care. Variations in carer positioning by the experts and health professionals in this study are attributable both to the context and nature of their interactions with carers and to the norms of each profession. Their divergent views support previous findings [13,31] that roles and settings are important contexts that can influence practices, and that we need to understand how health professionals other than physicians view shared decision making [6].

The views of community nurses were closest to those of carers, since they understood the DA as providing information and decision support that would allow carers to make service decisions, with or without health professional assistance. It may be that community nurses could see the DA as an adjunct and of assistance in their work because their home visiting role gives them access to the situated contexts that inform the reality of carers' circumstances. The counsellors, support workers and expert advisors, however, positioned themselves within a therapeutic model, in which they were experts and the carers were clients. This positioning influenced their beliefs about potentially confronting information, which was seen as something that needed to be provided (often in a filtered form) by experts when they judged the time to be right. The carer was viewed as either an un-well client or a non-coping client who needed to be helped. In expressing this positioning, counselors and support workers used language that was embedded in their occupational roles and reflected traditionally held views of patients within the medical model of care.

By contrast, carers themselves viewed the DA from a position of independence from health professionals, regarding it as a resource to provide them with information and skills in making service decisions. Like Wackerbarth [24], we found that many carers view themselves as competent in relation to services decision making and want information that will extend that competence. These data also resonate with Wackerbarth's [24] finding that uncertainty and lack of information makes it harder for carers to plan. It also suggests that any filtering of important realistic information about dementia can limit carers' agency, as it requires them to make relatively uninformed decisions. In practice, realistic information, however confronting, constitutes a positive resource for carers' deliberative judgments, and restricting this resource impedes their ability to weigh up all factors relevant to their circumstances.

The data further indicate that the beliefs of some health professionals may lead them to filter the provision of realistic information to carers, a finding also mentioned by Gravel, Legare and Graham [6]. The argument that carers' needs were being met or that carers needed protection from distressing information were part of a broader positioning of carers as passive service recipients. Such positioning maintains a status quo where health professionals control information [23] and the need to discuss difficult topics is avoided [37]. Our result fit with other findings that carers with knowledge of the facts of dementia demonstrate decreased rates of depression, more realistic expectations of outcomes, increased feelings of decision competence, and greater use of positive comparisons [25,26]. Hypotheses as to the causes of the association between information and anxiety for some carers include the possibilities that carers may become increasingly anxious if information is vague or not forthcoming [26] or that information augments carers' anticipation of loss [17]. Proctor et al. [26] suggest that the solution may be to provide more emotional support together with disturbing information. Independently of this debate, the empirical evidence indicates that carers want realistic information earlier in the disease [17].

Further research is needed to better understand why some health professionals limit information when evidence suggests that most carers' preference is for realistic and early information. Enhanced awareness of how the flow of decision support resources can facilitate or impede carers' decisional capacity is needed to inform the development and implementation of mechanisms such as DAs and innovative professional roles. While these results provide food for thought for service providers, we acknowledge the limitations of our study. Firstly, the fact that we did not collect these data specifically to consider the issue of carer positioning, nor directly observe carer/health professional interactions, meant that more in-depth information directly relating to on the issues raised in this paper was not elicited. We therefore recommend further research directly focused on this topic. Secondly our small convenience sample means that our results are not generalizable to any population of carers and health workers, and wider studies are needed. Nevertheless, as Myers [38] observes, small qualitative studies can provide a more personal understanding of phenomena that are valuable in themselves. In this tradition, we are confident that this study constitutes a modest but worthwhile contribution to the decision-making literature.

## Conclusion

Although many questions remain to be answered about how DAs work in different settings [39], this paper contributes to addressing an important research deficit. The beliefs and resulting practices associated with disparate health professions have been shown to lead to conflicting understandings of clients' information needs and the usefulness of decision support tools, with subsequent implications for decision aid implementation. We contend that if the primary health system is to embrace equal consumer participation in services, as advocated by recent analysts, carers must be explicitly positioned as competent agents who are able to make relevant evaluative judgments based on their own situations. Increased resources alone may not be sufficient to facilitate equality and balance in decision-making partnerships. Health workers' perspectives on client agency need to be addressed in greater depth if tools such as the DA are to overcome existing social barriers to resource flows. Although they specifically target counselors, Burnard's [30], page118 words of caution are apposite for all health service providers: "To withhold information and advice in order to satisfy a particular theory of how counseling should be practised may even be negligent", he warns. "To empower others, we must first rid ourselves of our own dogmas".

## Competing interests

The authors declare that they have no competing interests.

## Authors' contributions

CS generated the DA concept and with BL with assistance from JS, AR, TC, and JA carried out the qualitative development study. CS developed the first draft of the paper, and all other authors contributed to subsequent drafts of the paper. All authors read and approved the final manuscript.

## Pre-publication history

The pre-publication history for this paper can be accessed here:

http://www.biomedcentral.com/1472-6947/12/26/prepub
